# P-421. Impact of Rapid Methicillin-Resistant *Staphylococcus aureus* (MRSA) Nasal Polymerase Chain Reaction (PCR) Screening on Duration of Vancomycin (Vanc) Therapy for Suspected Pneumonia (PNA) in Adult Patients (Pts) Admitted to the Trauma Intensive Care Unit (TICU)

**DOI:** 10.1093/ofid/ofae631.622

**Published:** 2025-01-29

**Authors:** Shelby Brookshire, Mary Banoub, Olivia Randazza, Charles Hartis, James R Beardsley, John C Williamson, Seth Garner, Kristin Rebo, Elizabeth Palavecino, Vera P Luther, Werner Bischoff, Preston Miller, J Jason Hoth, Michael E DeWitt, Alex D Taylor

**Affiliations:** Atrium Health Wake Forest Baptist, Winston Salem, North Carolina; Atrium Health Wake Forest Baptist Medical Center, Winston Salem, North Carolina; Atrium Health Wake Forest Baptist, Winston Salem, North Carolina; Atrium Health Wake Forest Baptist, Winston Salem, North Carolina; Wake Forest University School of Medicine, Winston Salem, North Carolina; Atrium Health Wake Forest Baptist, Winston Salem, North Carolina; Atrium Health Wake Forest Medical Center, Winston Salem, North Carolina; Atrium Health Wake Forest Health, Winston Salem, North Carolina; Wake Forest School of Medicine, Winston Salem, North Carolina; Wake Forest University School of Medicine, Winston Salem, North Carolina; Wake Forest University School of Medicine, Winston Salem, NC; Wake Forest University School of Medicine, Winston Salem, North Carolina; Wake Forest School of Medicine, Winston Salem, North Carolina; Atrium Wake Forest Baptist Health/ Wake Forest University School of Medicine, Winston-Salem, North Carolina; Atrium Health Wake Forest Baptist, Winston Salem, North Carolina

## Abstract

**Background:**

At this institution on June 30, 2022, universal nasal decolonization with mupirocin ointment was replaced by nasal MRSA PCR assay screening at ICU admission to capture MRSA colonization and direct nasal decolonization. The aim of this study was to evaluate the impact of nasal MRSA PCR assay screening on duration of vanc for suspected PNA in adults admitted to the TICU.

**Table 1.**
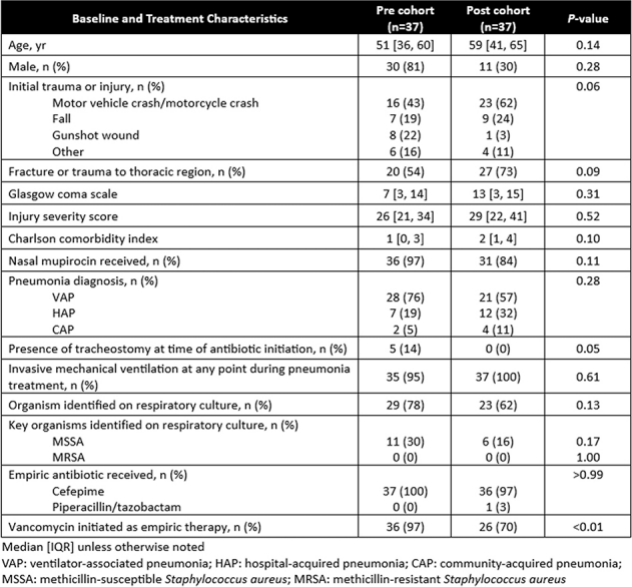
Baseline and Treatment Characteristics

**Methods:**

This single-center, retrospective cohort study compared pts from Jan - Jun 2022 (pre cohort) and Jan - Jun 2023 (post cohort). Pts ≥ 18 years of age admitted to the TICU that received empiric cefepime, meropenem, or piperacillin/tazobactam for suspected PNA documented on chest imaging were included. Pts who received vancomycin for a non-PNA indication, were previously treated for PNA during the admission, had structural lung disease, or had a pathogen identified on culture at the time of antibiotic (abx) initiation were excluded. The primary outcome was duration of vanc (hrs). Secondary outcomes included nasal MRSA PCR assay correlation with culture results from respiratory specimens, opportunities for vanc de-escalation based on negative nasal MRSA PCR assay within 36 hrs, ICU and hospital length of stay and mortality.

**Table 2.**
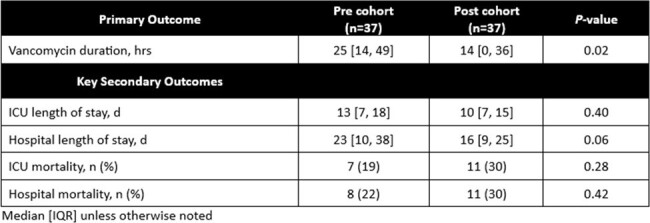
Primary and Secondary Outcomes

**Results:**

37 pts were included in each cohort. There were no significant differences in baseline characteristics (Table 1). 36 (97%) pts received vanc in the pre cohort compared to 26 (70%) pts in the post cohort (*P* < 0.01). The primary outcome (Table 2) of median vancomycin duration was 25 [14, 49] hrs in the pre cohort compared to 14 [0, 36] hrs in the post cohort (*P* = 0.02). Secondary outcomes are reported in Tables 2 and 3. The negative predictive value of the nasal MRSA PCR assay for PNA with MRSA was 100% in this study. MRSA was not identified on respiratory culture in either cohort.

**Table 3.**
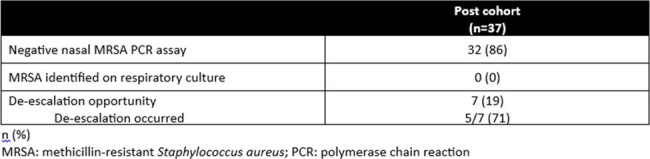
Opportunities for De-escalation (Post Cohort)

**Conclusion:**

Vanc usage and duration of therapy were significantly reduced after nasal MRSA PCR assay screening on ICU admission was implemented in the TICU. However, additional opportunities for improved stewardship of empiric initiation of vanc were identified based on the described vanc usage and lack of MRSA PNA.

**Disclosures:**

**John C. Williamson, PharmD**, Armata Pharmaceuticals: Grant/Research Support|Blue Collar Vaccines and Therapeutics: Board Member|Blue Collar Vaccines and Therapeutics: Ownership Interest|Paratek Pharmaceuticals: Grant/Research Support|ST Pharm Co, Ltd: Grant/Research Support

